# Association of osteoporosis and sarcopenia with fracture risk in transfusion-dependent thalassemia

**DOI:** 10.1038/s41598-023-43633-6

**Published:** 2023-09-29

**Authors:** Suttana Thavonlun, Natnicha Houngngam, Kanaungnit Kingpetch, Numphung Numkarunarunrote, Prangareeya Santisitthanon, Patinut Buranasupkajorn, Chatlert Pongchaiyakul, Pranee Sutcharitchan, Lalita Wattanachanya

**Affiliations:** 1 Division of Endocrinology and Metabolism, Department of Medicine, Faculty of Medicine, Chulalongkorn University, Bangkok, 10330 Thailand; 2 Excellence Center for Diabetes, Hormone, and Metabolism, King Chulalongkorn Memorial Hospital, Bangkok, 10330 Thailand; 3 Division of Nuclear Medicine, Department of Radiology, Faculty of Medicine, Chulalongkorn University, Bangkok, 10330 Thailand; 4 Division of Diagnostic Radiology, Department of Radiology, Faculty of Medicine, Chulalongkorn University, Bangkok, 10330 Thailand; 5 Division of Endocrinology and Metabolism, Department of Medicine, Faculty of Medicine, Khon Kaen University, Khon Kaen, 40002 Thailand; 6 Division of Hematology, Department of Medicine, Faculty of Medicine, Chulalongkorn University, Bangkok, 10330 Thailand

**Keywords:** Endocrinology, Ageing, Bone, Musculoskeletal system, Haematological diseases

## Abstract

Patients with transfusion-dependent thalassemia (TDT) have an increased risk of osteoporosis and fractures. They also have several potential factors associated with sarcopenia. There has been currently no study on sarcopenia and its association with falls and fractures in TDT. This study aims to determine the prevalence of and factors associated with osteoporosis, fragility fractures, and sarcopenia in adults with TDT. A cross-sectional study was conducted at the hematologic clinic at King Chulalongkorn Memorial Hospital, Bangkok, Thailand. Clinical data and laboratory testing were collected. Bone mineral density and morphometric vertebral fracture were assessed. Sarcopenia was defined using the 2014 and 2019 Asian Working Group for Sarcopenia (AWGS) criteria. We included 112 TDT patients aged 35.1 ± 12.5 years. The prevalence of osteoporosis was 38.4%. Fragility fractures were found in 20.5% of patients. Lower BMI (OR 0.29; 95% CI 0.12–0.72, *P* = 0.007) and hypogonadal state (OR 3.72; 95% CI 1.09–12.74, *P* = 0.036) were independently associated with osteoporosis. According to the 2014 AWGS criteria, the prevalence of overall sarcopenia and severe sarcopenia was 44.6% and 13.4%, respectively. Severe sarcopenia was strongly associated with fragility fractures (OR 4.59, 95% CI 1.21–17.46, *P* = 0.025). In conclusion, osteoporosis, fragility fractures, and sarcopenia were prevalent in adults with TDT. Severe sarcopenia was associated with fragility fractures. Early osteoporosis and sarcopenia screening and prevention may reduce fracture risk and its complications in these patients.

## Introduction

Thalassemia is one of the most common genetic disorders in the world characterized by ineffective erythropoiesis, hemolysis, and anemia due to defective globin production^1,2^. In the most severe form of thalassemia, transfusion-dependent thalassemia (TDT), patients require regular lifelong blood transfusions to survive^3,4^. Despite advances in transfusion and chelation therapy, high rates of morbidity and mortality from the disease itself as well as multiple end-organ dysfunctions caused by iron excess persist^2–4^.

Thalassemia bone disease (TBD) is characterized by bone pain, deformity, decreased bone mineral density (BMD), and fragility fractures. It has emerged as the major challenge in managing the growing morbidity burden associated with severe thalassemia phenotype^5^. Patients with TDT may experience pain as a result of fractures, which can develop over time^6^. In comparison to the general population, pain-related quality of life declines significantly with age^7^. Patients with more pain locations reported higher levels of anxiety and depression^8^.

The pathogenesis of TBD is incompletely understood but believed to be multifactorial, including childhood growth retardation, bone marrow expansion, iron and chelator toxicity, physical inactivity, low body weight, nutritional deficiencies, and hormonal deficiencies^4,9^. Depending on the study population, osteoporosis definition, and co-morbidities, the prevalence of osteoporosis in adults with TDT has been reported to range from 17.6 to 74.6%^10–13^. Compared to controls, adolescents and young adults with TDT had lower trabecular volumetric BMD, cortical area, cortical thickness, and periosteal circumference^14^. A recent meta-analysis found a higher prevalence of fractures in TDT (18%, 95% confidence interval (CI) 16–19%) than in non-TDT (7%, 95% CI 4–10%)^15^. Although osteoporosis was strongly associated with increased risk of fracture in postmenopausal women and aging men, it was unknown whether decreasing BMD predicted fracture in TDT^14,16–19^.

Sarcopenia is a progressive clinical syndrome characterized by general loss of skeletal muscle mass and impaired muscle function^20,21^. Muscle and bone are anatomically and functionally connected. The evidence associating sarcopenia, low BMD, and fracture with an elevated risk of mortality and morbidity has been widely characterized in the general population^22^. TDT patients presented various potential risk factors for sarcopenia, including low body weight, poor nutrition, limited physical activity, and chronic inflammatory stage^2,4,9^. As a result, it is critical to investigate sarcopenia in these patients.

Currently, there have been no studies examining the prevalence of sarcopenia and its associated negative health outcomes in TDT patients. It is also unclear whether sarcopenia is associated with TDT-related falls and fractures. Therefore, the current study aimed to determine the prevalence of osteoporosis and sarcopenia in adults with TDT and examine their association with fracture risk. Our findings will contribute to a greater comprehension of the pathogenesis of bone abnormalities in TDT, which may aid in the development of fracture prevention strategies in order to decrease fracture risks and their complications.

## Results

### Characteristics of the study population

A total of 112 patients were included in the final analysis. Study recruitment flow is summarized in Supplementary Fig. 1. There were 66 women and 46 men with a ratio of 1.4 to 1. The mean age and BMI were 35.1 ± 12.5 years and 19.9 ± 2.7 kg/m^2^ respectively. One-third of them (9 men and 27 women) had hypogonadism. All of the men and 14 of the 27 women had secondary hypogonadism, and 13 women reached natural menopause. All of the patients received regular blood transfusions and iron chelation therapy, and 41.1% underwent splenectomy. Seven patients (6.3%) had a history of falls in the past year. Vitamin D deficiency was prevalent among women (50.9%). Endocrinopathies included low IGF-1 levels (33.9%), subclinical/secondary hypothyroidism (32.2%), and secondary hypogonadism (21.4%). Men and women with hypogonadism had significantly lower IGF-1 levels than eugonadal patients. The demographic, clinical, and laboratory characteristics of the study population are shown in Table 1.

**Table 1:** Demographic, clinical and laboratory characteristics of the study population, stratified by sex and gonadal status.

	Total (N = 112)	Female			Male		
		All (N = 66)	Eugonadal (N = 39)	Hypogonadal (N = 27)	All (N = 46)	Eugonadal (N = 37)	Hypogonadal (N = 9)
Demographic and clinical characteristics							
Age, year	35.1 ± 12.5	38.2 ± 13.2	33.0 ± 9.8	45.7 ± 13.9**	30.8 ± 10.2	28.6 ± 9.5	39.8 ± 8.6**
BMI, kg/m^2^	19.9 ± 2.7	20.2 ± 2.6	20.1 ± 2.2	20.5 ± 3.2	19.5 ± 2.9	19.3 ± 2.7	20.2 ± 3.7
β/E thalassemia (%)	100 (89.3)	57 (86.3)	33 (84.6)	24 (88.9)	43 (93.5)	35 (94.6)	8 (88.9)
Smoking, n (%)	5 (4.5)	2 (3.0)	0 (0.0)	2 (7.4)	3 (6.5)	3 (8.1)	0 (0.0)
Alcohol drinking (%)	11 (9.8)	6 (9.1)	3 (7.7)	3 (11.1)	5 (10.9)	4 (10.8)	1 (11.1)
History of falls in the past year (%)	7 (6.3)	5 (7.6)	3 (7.7)	2 (7.4)	2 (4.3)	0 (0.0)	2 (22.2)*
Splenectomy (%)	46 (41.1)	23 (34.8)	13 (33.3)	10 (37.0)	23 (50.0)	18 (48.6)	5 (55.6)
Blood transfusion, unit/years	24.7 ± 9.8	24.5 ± 9.4	24.8 ± 8.4	24.0 ± 10.9	25.0 ± 10.5	23.5 ± 9.7	31.1 ± 11.9
Iron chelation (%)							
Deferiprone	62 (55.4)	33 (50.0)	18 (46.2)	15 (55.6)	29 (63.0)	26 (70.3)	3 (33.3)*
Deferoxamine	3 (2.7)	2 (3.0)	0 (0.0)	2 (7.4)	1 (2.2)	1 (2.7)	0 (0.0)
Deferasirox	29 (25.9)	20 (30.3)	12 (30.8)	8 (29.6)	9 (19.6)	6 (16.2)	3 (33.3)
Laboratory findings							
Pre transfusion Hb, g/dL	7.96 ± 1.53	7.7 ± 1.4	7.58 ± 1.59	7.90 ± 1.06	8.32 ± 1.64	8.54 ± 1.59	7.42 ± 1.64
Ferritin, ng/mL	1945 (1019–4380)	2228 (1135–4503)	2270 (833–4626)	2204 (1340–4461)	1545 (995–4248)	1404 (851–3291)	4139 (1695–9669)*
Ferritin ≥ 1000 ng/mL (%)	85 (75.9)	50 (75.8)	27 (69.2)	23 (85.2)	35 (76.1)	26 (70.3)	9 (100.0)
FPG, mg/dL	96 ± 32	98 ± 40	98 ± 47	100 ± 27	92 ± 15	88 ± 8	110 ± 24*
Creatinine, mg/dL	0.52 (0.43–0.63)	0.50 (0.38–0.56)	0.49 (0.38–0.56)	0.52 (0.42–0.61)	0.58 (0.47–0.67)	0.58 (0.49–0.67)	0.55 (0.37–0.71)
Albumin, g/dL	4.3 ± 0.4	4.3 ± 0.4	4.4 ± 0.3	4.2 ± 0.4*	4.4 ± 0.4	4.4 ± 0.4	4.2 ± 0.4
Calcium, mg/dL	8.9 ± 0.4	8.8 ± 0.4	8.8 ± 0.4	8.9 ± 0.4	8.9 ± 0.3	8.9 ± 0.3	8.9 ± 0.4
25(OH)D, ng/mL	21.2 ± 7.6	19.9 ± 7.4	19.8 ± 7.1	20.3 ± 8.0	22.8 ± 7.6	23.7 ± 7.4	19.0 ± 7.7
Intact PTH, pg/mL	36 (29–48)	40 (32–52)	38 (31–48)	40 (32–64)	34 (26–39)	34 (27–39)	35 (20–41)
IGF-1, ng/mL	62 (39–100)	67 (39–94)	76 (46–101)	44 (28–87)**	60 (39–118)	72 (47–128)	36 (17–47)**
FSH, IU/L	–	9.2 (3.2–36.7)	7.2 (3.3–10.3)	12.9 (2.5–40.9)	4.0 (2.8–5.5)	4.7 (3.1–5.6)	1.3 (0.7–4.2)*
LH, IU/L	–	7.8 (2.4–14.4)	8.8 (5.7–11.3)	7.8 (1.1–25.5)	6.8 (4.1–9.5)	7.1 (5.2–10.1)	2.2 (1.0–6.3)**
Estradiol, pg/mL	–	13.8 (5.0–45.7)	90.8 (62.2–136.1)	6.2 (5.0–19.2)**	–	–	–
TT, nmol/L	–	–	–	–	27.7 (19.8–38.8)	32.2 (22.1–42.3)	3.2 (0.7–11.8)**
Calculated FT, %	–		–	–	0.23 (0.28–0.49)	0.46 (0.37–0.51)	0.02 (0.004–0.12)**
SHBG, nmol/L	–	–	–	–	79.8 ± 36.0	71.5 ± 30.3	113.9 ± 39.3**
CTX, ng/mL	0.62 (0.44–0.84)	0.59 (0.39–0.82)	0.49 (0.36–0.73)	0.66 (0.44–0.96)	0.62 (0.48–0.92)	0.68 (0.49–0.92)	0.55 (0.38–0.79)
P1NP, ng/mL	54.8 (41.4–79.9)	55.6 (40.7–78.8)	47.7 (39.7–66.5)	67.1 (49.1–88.8)**	54.5 (43.2–90.7)	54.4 (46.2–91.6)	59.8 (36.8–91.1)
Endocrinopathies							
Diabetes mellitus (%)	6 (5.4)	4 (6.1)	2 (5.1)	2 (7.4)	2 (4.3)	0 (0.0)	2 (22.2)*
Vitamin D deficiency (%)	57 (50.9)	40 (60.6)	23 (59.0)	17 (63.0)	17 (37.0)	12 (32.4)	5 (55.6)
Hypoparathyroidism (%)	1 (0.9)	0 (0.0)	0 (0.0)	0 (0.0)	1 (2.2)	0 (0.0)	1 (11.1)
Secondary/subclinical hypothyroidism (%)	36 (32.2)	22 (33.3)	13 (33.4)	9 (33.3)	14 (30.4)	8 (21.6)	6 (66.7)**
Secondary hypogonadism (%)	24 (21.4)	15 (22.7)	0 (0.0)	15 (55.6)**	9 (19.6)	0 (0.0)	9 (100.0)**
Low IGF-1 level (%)	38 (33.9)	16 (24.2)	7 (17.9)	9 (33.3)**	22 (47.8)	14 (37.8)	8 (88.9)**

Values are expressed as mean ± SD when normally distributed, as median (IQR) when not normally distributed, and as percentages when categorical.

*BMI* body mass index, *Hb* hemoglobin, *FPG* fasting plasma glucose, *25(OH)D* 25-hydroxyvitamin D, *PTH* parathyroid hormone, *IGF-1* insulin-like growth factor 1, *FSH* follicle stimulating hormone, *LH* luteinizing hormone, *TT* total testosterone, *FT* free testosterone, *SHBG* sex hormone binding globulin, *CTX* C-terminal cross-linking telopeptide of type I collagen, *P1NP* procollagen type 1 N-terminal propeptide.

*P* values by unpaired t-test, Mann–Whitney U, and Chi-square or Fisher’s exact test for parametric, non-parametric, and categorical variables respectively. *P < 0.05, **P < 0.01 versus Eugonadal.

### Prevalence of osteoporosis, fragility fracture, and sarcopenia in adults with TDT

Fifty-eight percent of the patients had BMD below the expected range for their age. Based on the BMD T-score, 55.4% of the patients had osteopenia and 38.4% had osteoporosis. The majority of patients had either osteoporosis at both the lumbar spine and the hip (48.8%) or only at the lumbar spine (37.2%), while a minority had isolated hip osteoporosis (14%). At all sites, osteoporosis was prevalent in hypogonadal patients of both sexes. The prevalence of fragility fracture and morphometric VF were 20.5% (23/112) and 8.0% (9/112), respectively. The majority of patients had a single fracture with the lumbar spine having the highest prevalence, followed by the forearms, humerus, femurs, and clavicle. Women tended to have fractures more than men, and although not statistically significant, hypogonadal women had more fractures than eugonadal women (Table 2).

**Table 2:** Bone mineral density, morphometric vertebral fracture, and all fragility fracture, stratified by sex and gonadal status.

	Total (N = 112)	Female			Male		
		All (N = 66)	Eugonadal (N = 39)	Hypogonadal (N = 27)	All (N = 46)	Eugonadal (N = 37)	Hypogonadal (N = 9)
BMD T-score							
Lumbar spine	− 2.23 ± 1.15	− 2.12 ± 1.03	− 1.98 ± 1.06	− 2.32 ± 0.97	− 2.38 ± 1.30	− 2.23 ± 1.28	− 2.98 ± 1.31
Total hip	− 1.28 ± 1.08	− 1.13 ± 1.00	− 0.87 ± 1.00	− 1.51 ± 0.88**	− 1.50 ± 1.16	− 1.41 ± 1.19	− 1.89 ± 0.99
Femoral neck	− 1.51 ± 1.03	− 1.64 ± 0.97	− 1.34 ± 0.96	− 2.07 ± 0.84**	− 1.33 ± 1.09	− 1.21 ± 1.09	− 1.82 ± 1.06
BMD Z-score, n (%)							
≤ − 2.0	58 (51.8)	32 (48.5)	19 (48.7)	13 (48.1)	26 (56.5)	20 (54.1)	6 (66.7)
> − 2.0	54 (48.2)	34 (51.5)	20 (51.3)	14 (51.9)	20 (43.5)	17 (45.9)	3 (33.3)
BMD T-score, n (%)							
≤ − 2.5	43 (38.4)	27 (40.9)	10 (25.6)	17 (63.0)**	16 (34.8)	10 (27.0)	6 (66.7)*
< − 1.0 to > − 2.5	62 (55.4)	35 (53.0)	26 (66.7)	9 (33.3)**	27 (58.7)	24 (64.9)	3 (33.3)
≥ − 1.0	7 (6.3)	4 (6.1)	3 (7.7)	1 (3.7)	3 (6.5)	3 (8.1)	0 (0.0)
Morphometric VF, n (%)	9 (8.0)	7 (10.6)	2 (5.1)	5 (18.5)	2 (4.3)	1 (2.7)	1 (11.1)
All fragility fracture, n (%)	23 (20.5)	16 (24.2)	8 (20.5)	8 (29.6)	7 (15.2)	6 (16.2)	1 (11.1)

Values are expressed as mean ± SD or as percentages when categorical. *P* values by unpaired t-test and Chi-square test for parametric and categorical variables respectively.

*BMD* bone mineral density, *VF* vertebral fracture.

*P < 0.05, **P < 0.01 versus Eugonadal.

In this study, 98.2% of the patients had low skeletal muscle mass. According to the 2014 AWGS criteria, 22.3% and 17.9% had low gait speed and low handgrip strength, respectively. The prevalence of overall sarcopenia was 44.6% (sarcopenia 31.2%, severe sarcopenia 13.4%). Using the 2019 revised AWGS criteria, the prevalence of overall sarcopenia and severe sarcopenia increased to 61.6% and 24.1%, respectively (Table 3). About half of sarcopenic patients had osteoporosis and vice versa (Supplementary Fig. 2).

**Table 3:** Prevalence and components of sarcopenia and severe sarcopenia based on the 2014 and 2019 Asian Working Group for Sarcopenia (AWGS) criteria.

	Total (N = 112)	Female			Male		
		All (N = 66)	Eugonadal (N = 39)	Hypogonadal (N = 27)	All (N = 46)	Eugonadal (N = 37)	Hypogonadal (N = 9)
The 2014 AWGS criteria							
Overall sarcopenia, n (%)	50 (44.6)	34 (51.5)	21 (53.8)	13 (48.1)	16 (34.8)	13 (35.1)	3 (33.3)
Sarcopenia, n (%)	35 (31.2)	25 (37.9)	18 (46.1)	7 (25.9)	10 (21.7)	9 (24.3)	1 (11.1)
Severe sarcopenia, n (%)	15 (13.4)	9 (13.6)	3 (7.7)	6 (22.2)	6 (13.0)	4 (10.8)	2 (22.2)
The 2019 AWGS criteria							
Overall sarcopenia, n (%)	69 (61.6)	43 (65.2)	25 (64.1)	18 (66.7)	26 (56.5)	21 (56.7)	5 (55.5)
Sarcopenia, n (%)	42 (37.5)	25 (37.9)	15 (38.5)	10 (37.0)	17 (37.0)	15 (40.5)	2 (22.2)
Severe sarcopenia, n (%)	27 (24.1)	18 (27.3)	10 (25.6)	8 (29.7)	9 (19.5)	6 (16.2)	3 (33.3)

### Factors associated with osteoporosis, fragility fractures, and sarcopenia

Results from the univariate analysis revealed that age, BMI, a history of falls in the past year, serum ferritin level, hypogonadal state, CTX, P1NP, IGF-1 level, and severe sarcopenia were associated with osteoporosis. In multivariate analysis, however, only BMI (OR 0.29; 95% CI 0.12–0.72, *P* = 0.007) and hypogonadal state (OR 3.72; 95% CI 1.09–12.74, *P* = 0.036) were independently associated with osteoporosis (Supplementary Table S1).

According to the 2014 AWGS criteria, a history of falls in the past year, IGF-1 levels, osteoporosis, and severe sarcopenia were associated with fragility fracture. In multivariate analysis, severe sarcopenia (OR 4.59; 95% CI 1.21, 17.46, *P* = 0.025) and IGF-1 levels (OR 0.98; 95% CI 0.96, 0.99, *P* = 0.023) were independently associated with fragility fracture (Table 4). In addition, age (*P* = 0.032) and deferasirox usage (*P* = 0.031) were significantly associated with overall sarcopenia, while a history of falls in the past year (*P* = 0.032) was associated with severe sarcopenia. However, these associations were not found significant in multivariate analyses (Table 5).

**Table 4:** Factors associated with fragility fracture.

Variables	Univariate analysis		Multivariate analysis	
	OR (95% CI)	*P* value	OR (95% CI)	*P* value
Age per 10 years	1.40 (0.96, 2.06)	0.083	0.79 (0.46, 1.37)	0.416
Female sex	1.78 (0.67, 4.76)	0.248		
BMI per 5 kg/m^2^	0.84 (0.38, 1.86)	0.670		
Hypogonadal stage	1.48 (0.57, 3.82)	0.422		
History of falls in the past year	6.04 (1.25, 29.22)	0.025	2.39 (0.39, 14.86)	0.348
Current smoking	0.00 (0.00, 0.00)	0.999		
Alcohol drinking > 1 unit/day	0.85 (0.17, 4.22)	0.839		
Splenectomy	1.41 (0.56, 3.56)	0.461		
Iron chelating agents				
Deferiprone	1.33 (0.52, 3.39)	0.552		
Deferasirox	1.73 (0.64, 4.64)	0.278		
Pre transfusion Hb, g/dL	1.05 (0.75, 1.47)	0.768		
Ferritin per 1000 ug/L	1.04 (0.89, 1.20)	0.614		
25(OH)D, ng/mL	1.04 (0.98, 1.10)	0.198	1.06 (0.99, 1.13)	0.097
IGF-1, ng/mL	0.98 (0.97, 0.99)	0.013	0.98 (0.96, 0.99)	0.023
Osteoporosis	2.56 (1.00, 6.51)	0.049	1.49 (0.49, 4.58)	0.487
Sarcopenia stage (the 2014 AWGS)				
Sarcopenia	0.52 (0.19, 1.45)	0.209		
Severe sarcopenia	4.43 (1.41, 13.95)	0.011	4.59 (1.21, 17.46)	0.025

Logistic regression was used to assess factors associated with fracture. *BMI* body mass index, *Hb* hemoglobin, *25(OH)D* 25-hydroxyvitamin D, *IGF-1* insulin-like growth factor 1, *AWGS* Asian Working Group for Sarcopenia.

**Table 5:** Factors associated with sarcopenia diagnosed by the 2014 Asian Working Group for sarcopenia criteria.

Variables	Overall sarcopenia				Severe sarcopenia			
	Univariate		Multivariate		Univariate		Multivariate	
	OR (95% CI)	*P* value	OR (95% CI)	*P* value	OR (95% CI)	*P* value	OR (95% CI)	*P* value
Age per 10 years	1.44 (1.03, 1.99)	0.032	1.48 (0.89, 2.45)	0.127	1.26 (0.80, 1.97)	0.321		
Female sex	1.99 (0.92, 4.33)	0.081	1.86 (0.76, 4.54)	0.176	1.05 (0.35, 3.19)	0.928		
BMI per 5 kg/m^2^	0.59 (0.31, 1.12)	0.108	0.65 (0.31, 1.35)	0.247	0.58 (0.21, 1.63)	0.305		
Hypogonadal stage	0.99 (0.45, 2.19)	0.977			2.82 (0.93, 8.51)	0.066	2.02 (0.60, 6.75)	0.256
History of falls in the past year	8.32 (0.97, 71.57)	0.054	4.88 (0.48, 49.53)	0.180	5.81 (1.16, 29.17)	0.032	5.55 (0.86, 35.97)	0.072
Splenectomy	1.68 (0.78, 3.59)	0.182	2.66 (0.97, 7.24)	0.056	1.30 (0.44, 3.88)	0.637		
Iron chelating agents								
Deferiprone	0.68 (0.32, 1.43)	0.307			0.49 (0.16, 1.48)	0.205		
Deferasirox	2.61 (1.09, 6.23)	0.031	1.56 (0.58, 4.21)	0.378	2.98 (0.97, 9.14)	0.056	2.23 (0.66, 7.55)	0.197
Ferritin per 1000 ug/L	1.12 (0.98, 1.27)	0.098	1.09 (0.93, 1.28)	0.282	1.15 (0.99, 1.34)	0.078	1.05 (0.87, 1.26)	0.646
Pre transfusion Hb, g/dL	1.03 (0.78, 1.36)	0.855			1.04 (0.69, 1.55)	0.851		
25(OH)D, ng/mL	1.00 (0.95, 1.05)	0.946			1.02 (0.95, 1.09)	0.638		
Diabetes mellitus	0.23 (0.03, 2.06)	0.190	0.29 (0.03, 3.09)	0.311	1.31 (0.14, 12.09)	0.809		
IGF-1, ng/mL	0.99 (0.99, 1.00)	0.199	1.00 (0.99, 1.02)	0.566	1.00 (0.99, 1.01)	0.808		

Logistic regression was used to assess factors associated with sarcopenia. *BMI* body mass index, *Hb* hemoglobin, *25(OH)D* 25-hydroxyvitamin D, *IGF-1* insulin-like growth factor 1.

## Discussion

The present study evaluated the prevalence of osteoporosis and fragility fractures, focusing on the prevalence of sarcopenia and its associated risk factors in TDT. Half of the patients in our study had low bone mass for their age, and 38.4% had osteoporosis as defined by BMD T-score ≤ − 2.5. The overall prevalence of fragility fractures was 20.5%. Furthermore, we demonstrated that nearly all patients had low skeletal muscle mass with almost half having sarcopenia. We also found that severe sarcopenia was strongly associated with fragility fractures.

The prevalence of osteoporosis and fracture in TDT patients was highly heterogeneous, depending on patient characteristics, comorbidities, diagnostic criteria of osteoporosis, assessment method, definition of fracture, and sample size. Our findings were consistent with previous reports^10,12^, which found that approximately half of adults with TDT had BMD values below the expected range for their age. In a recent study conducted in Iran^13^, the prevalence of low BMD was found to be 74.6%, with half of the participants having hypogonadism, compared to 32.1% in our study. In contrast, a 17.6% rate of osteoporosis was reported in a study that omitted TDT patients with hypogonadism or menopause^11^. All studies, including our study, demonstrated a higher prevalence of osteoporosis at the lumbar spine than the hip region, regardless of gender or gonadal status.

The prevalence of fracture among adults with TDT was also highly variable, ranging from 11.6^23^ to 55%^19^. However, most fracture data were collected via a self-reported questionnaire and/or medical record with each method having different types of possible bias. Some studies included traumatic fractures in reported total fractures, which could overestimate the prevalence of fragility fractures. In our study, fracture data was confirmed and accurately reported through an in-person interview following a review of medical records. Asymptomatic VFs were also evaluated with spinal X-rays, and hands, finger, foot, ankle, skull and facial bone fractures were excluded. We feel our study more accurately reflected the occurrence of fragility fractures among these patients compared to most previous studies.

The underlying mechanisms that lead to decreased bone mass and increased fracture risk in TDT remain unknown, but it is believed that they are complex and involve both traditional and thalassemia-specific risk factors^9^. TDT patients are likely to have several traditional risk factors for low BMD/fracture, including physical inactivity, low body weight, and nutritional deficiencies^4,9,24^. Several nontraditional factors have been investigated, including genetic factors^25,26^, marrow expansion^27^, iron toxicity^28,29^, and hormonal deficiencies^30,31^. The present study has confirmed the association between hypogonadism and osteoporosis in TDT patients, which has been reported in several studies^30,32–34^. We found that the IGF-1 level was associated with osteoporosis, however, the association did not attain statistical significance in a multivariate analysis. The study findings indicated that individuals with hypogonadism had significantly lower IGF-1 levels than those with eugonadism. A negative correlation was also observed between IGF-1 levels and fracture. It is noteworthy to mention that the inability to attain maximum bone mass^19,31^ leading to potential fractures in TDT patients can be attributed to both hypogonadism and GH deficiency.

In postmenopausal osteoporosis, the relationship between BMD measured by DXA and fracture is well-established^35,36^. The effect of BMD on fracture in TDT was controversial^23,37^. We found no association between BMD and fracture, which could be explained by the fact that TBD is a composite of several factors, many of which cause significant bone microarchitecture deterioration, but are not entirely reflected by BMD.

Vitamin D plays an important role in calcium and skeletal homeostasis, and vitamin D deficiency results in secondary hyperparathyroidism, bone loss, and mineralization defects^38^. Despite the high prevalence of low vitamin D status among TDT patients^39–42^, the contribution of vitamin D to TBD has not been shown. We found that half of our patients had vitamin D deficiency, a rate comparable to that of the general Thai population^43^. However, no association was found between vitamin D status and fracture. The majority of our patients had 25(OH)D levels greater than 12 ng/dL, which was unlikely to result in osteomalacia. In addition, there was no correlation between low 25(OH)D and high intact parathyroid hormone, suggesting that they may have relative hypoparathyroidism. These factors may partially explain why vitamin D deficiency was not associated with bone loss and/or fracture.

Sarcopenia is an age-related disease, characterized by a general loss of skeletal muscle mass and impaired muscle function, and is associated with a wide range of adverse health outcomes, including falls and fractures^44^. In addition to elderly populations, sarcopenia was substantially more common in various patient categories with the prevalence ranging from 18% in diabetic individuals to 66% in patients with unresectable esophageal cancer. A high prevalence of sarcopenia has also been found in patients with renal and liver disease who require surgery, as well as in individuals with various site-specific malignancies^45^. According to the 2014 AWGS definition, we found that sarcopenia was prevalent in young to middle-aged adults with TDT (44.6%), and that 30% of these individuals had severe sarcopenia. Even though there have been no comparative studies of sarcopenia in patients with thalassemia, the above prevalence was much higher than reported among community-dwelling older Thai people (ranging from 10 to 30%)^46–48^, indicating that TDT accelerated the development of sarcopenia. We suggested that physicians should be aware of the risk of sarcopenia in TDT patients, not just the elderly, but also the middle-aged.

Several mechanisms, including aging, oxidative stress, inflammation, mitochondrial dysfunction, and apoptosis may be involved in the pathogenesis of sarcopenia^44^. Underlying mechanisms leading to sarcopenia in TDT have not been adequately studied. We believe that they are multifactorial, including both risk factors found in the general elderly population and risk factors specific to thalassemia.

Age-related iron accumulation in skeletal muscles causes muscle atrophy and is likely associated with oxidative damage and mitochondrial dysfunction, according to animal studies^49^. Recent clinical data have consistently suggested a correlation between iron accumulation and sarcopenia. Kim et al.^50^ demonstrated that serum ferritin levels were substantially associated with sarcopenia in middle-aged and elderly Korean women. Patients with elevated serum ferritin levels had a twofold increased risk of sarcopenia compared to those with normal serum ferritin levels. Another study by Perna et al.^51^ also found that elderly patients with sarcopenia had significantly elevated serum ferritin levels and serum inflammatory markers. These findings suggested that excess iron may play a role in the development of sarcopenia in TDT. The presence of chronic, severe anemia may contribute to sarcopenia in TDT patients. In a previous study involving middle-aged to elderly individuals^52–54^, a negative association between hemoglobin level and sarcopenia was observed. In one report, a 1 g/dL increase in hemoglobin was associated with a 5% reduction in the risk of sarcopenia (OR 0.95, 95% CI 0.90–0.98)^53^. In the general population, an association between hormonal deficiencies, such as vitamin D deficiency, hypogonadism, growth hormone deficiency, and sarcopenia has been described^55^. However, there have been few studies investigating the effect of these factors on muscle mass and/or function in TDT patients^56^.

In our study, we found no significant association between sarcopenia (based on either the 2014 or 2019 AWGS criteria) and age, BMI, vitamin D level, IGF-1 level, hypogonadism, pre-transfusion hemoglobin level, or serum ferritin level. A relatively small sample size and distinctions in the study population may have resulted in insufficient power to detect any associations in the multivariate analyses.

In the general population, the effect of decreased muscle mass and strength on fall risk, BMD, and fractures has been well described^57–59^. In one retrospective cohort of adults with TDT, there was a positive correlation between skeletal muscle mass and BMD^60^. No association between sarcopenia and BMD was demonstrated in our study, however, we found that a significant proportion of TDT patients with osteoporosis had sarcopenia and vice versa. Therefore, sarcopenia should be routinely assessed in TDT patients, especially for those with osteoporosis.

A recent meta-analysis of prospective studies in older adults found that sarcopenic individuals had a significantly higher risk of falls (OR 1.89; 95% CI 1.33–2.68,* P* < 0.001) and fractures (OR 1.71; 95% CI 1.44–2.03, *P* = 0.011) than non‐sarcopenic individuals^61^. Although we found no association between sarcopenia and falls in TDT, patients with severe sarcopenia, according to the 2014 AWGS criteria had a 4.6-fold increased risk of fragility fracture (OR 4.59, 95% CI 1.21–17.46, *P* = 0.025).

According to our knowledge, this is the first study to report the prevalence and risk factors of sarcopenia in adults with TDT. Our findings highlighted the significance of sarcopenia in TDT, which was not only common and occurred at a younger age, but was also associated with fracture. Although there is limited evidence to support sarcopenia as a predictor of osteoporotic fractures, it is widely accepted that sarcopenia and osteoporosis/fracture are strongly connected. We consider that assessing sarcopenia should be incorporated of any fracture prevention strategy in TDT patients, particularly those with osteoporosis. In the absence of specific sarcopenia medications, interventions known to improve sarcopenia in the general population, such as exercise, protein supplements, and adequate vitamin D levels, may have an effect on bone mass and should be encouraged in these individuals. A longitudinal investigation on the outcome of sarcopenia in TDT patients might be beneficial.

This study contains some limitations. First, the participants were recruited from a hematology clinic at a university hospital in Thailand, where the majority of patients had received early and regular blood transfusions and chelation therapy and were extensively monitored and regularly followed up. Findings cannot necessarily be generalized to other settings where resources are more limited. Second, most participants in the study were young to middle-aged adults, so the study results also should not be generalized to children, adolescents or older adults until further study. Third, the prevalence of osteoporosis may be exaggerated if the T-score is used to classify BMD for all participants, as some of them may not have reached their peak bone mass. However, the proportion of patients with low BMD by Z-score was comparable between adults under and over 30 years of age, indicating that low BMD is likely to persist in younger adults as they age. Fourth, we lacked information on dietary intake, physical activity, and inflammatory markers, which could be additional risk factors for sarcopenia. In addition, our study did not assess other potential consequences of sarcopenia, such as physical disabilities, depression, and decreased quality of life. Last, as a cross-sectional investigation, we were unable to provide support for a causal relationship between the associated factors and the outcomes.

## Conclusion

Osteoporosis, fragility fractures, and sarcopenia were prevalent among adult patients with TDT. Severe sarcopenia was strongly associated with fragility fracture. Screening, prevention, and treatment strategies for osteoporosis and sarcopenia may reduce fracture risk and its complications.

## Materials and methods

### Study design and participants

A cross-sectional study was conducted between August 2020 and November 2020. One hundred and fifty-nine TDT patients over 18 years of age who attended the hematology clinic of the King Chulalongkorn Memorial Hospital, Bangkok, Thailand, were invited to participate in this study. Participants with a known history of bone marrow transplantation, cancer, inflammatory bowel disease, being on anti-osteoporotic drugs or medications affecting bone metabolism, and/or being hospitalized were excluded. A total of 112 patients who met the inclusion criteria were enrolled.

The study was reviewed and approved by the Chulalongkorn University Faculty of Medicine’s Institutional Review Board in Bangkok, Thailand (IRB number 126/63). All study participants provided written informed consent before any procedures were performed. All procedures were conducted following applicable rules and guidelines. The trial registration number was TCTR 20201008006.

### Clinical data collection

At the time of study enrollment, the demographic and clinical information of all participants were collected from an electronic medical record system and interviewed by trained study personnel. A structured questionnaire was used to collect data, including a history of thalassemia, reproduction, and traditional osteoporosis and fracture risk history. We measured anthropometric variables, including weight and current height. The data collection was performed at the same visit as the blood sample collection and BMD measurement or within a month.

After at least 8 h of fasting, whole blood samples were collected, and serum and plasma were stored at − 80 °C until analysis. Complete blood count, ferritin, calcium, phosphorus, albumin, creatinine, liver function tests, fasting plasma glucose, insulin-like growth factor-1 (IGF-1), intact parathyroid hormone (ICMA; intact PTH assays Roche Elecsys®), serum 25-hydroxyvitamin D (25(OH)D) levels (CLIA; DiaSorin LIAISON®), free thyroxin (T4) and thyroid stimulating hormone (TSH) (ECLIA; Cobas e601 analyzer, Roche Diagnostics) were measured in all participants. A fasting plasma glucose level of ≥ 126 mg/dL was used to define diabetes. Subclinical hypothyroidism was defined as a TSH level above the upper limit of the reference range and a normal level of free T4, and overt hypothyroidism was defined as a free T4 level below the lower limit of the reference range with any TSH concentration. In this study, low IGF-1 was defined as IGF-1 less than the lower limit of normal IGF-1 level of that age. Age-specific reference ranges of IGF-1 levels were provided by SIEMENS (IMMULITE® 2000 systems). For the 25(OH)D assay, the intra- and interassay coefficients of variation were less than 6%. Vitamin D deficiency was defined as serum 25(OH)D levels of < 20 ng/mL^62^. Bone turnover markers including serum procollagen type 1 N-terminal propeptide and C-terminal cross-linking telopeptide of type I collagen were measured using a chemiluminescence immunoassay (Roche Diagnostics, Mannheim, Germany) in an Elecsys 2010 machine.

Serum FSH, LH, total testosterone, and sex hormone binding globulin levels were measured in all men. Men with calculated free testosterone concentration of < 0.245 nmol/L based on Vermeulen’s formula were defined as hypogonadism^63^. In women with irregular menstruation, FH, LH, and estradiol levels were measured. Secondary hypogonadism was diagnosed in women with irregular or absent menstruation for at least 12 months with serum estradiol concentrations < 50 pg/mL and inappropriately low or normal serum FSH concentrations. Woman over the age of 45 years who had not had a menstrual period for a year and had FSH levels > 30 mIU/mL was diagnosed as menopause^64^.

### Bone mineral density measurement

BMD at the lumbar spine, femoral neck, and total hip were measured using dual energy X-ray absorptiometry (DXA) on a QDR 4500 bone densitometer (Hologic, Inc., Bedford, MA). BMD Z- and T-scores were calculated using the manufacturer-supplied reference databases for Asian populations. According to the IOF Committee of Scientific Advisors Working Group on Osteoporosis Pathophysiology 2012^65^, they recommended using BMD T-score according to the WHO operational definition of osteoporosis in young adults with a chronic disorder or taking medications known to affect bone metabolism, unless it appears that he/she is still growing. In this study, osteoporosis was defined as a T-score of ≤  − 2.5 standard deviation (SD), whereas osteopenia was defined as a T-score between − 1 and − 2.5 SD below the young adult mean value. BMD Z-scores were also calculated. Individuals with a Z-score ≤  − 2.0 SD were defined as having low bone mass compared to a healthy person of the same age and gender^66^.

### Fragility fracture and morphometric vertebral fracture assessment

A fragility fracture is defined as a fracture sustained from force comparable to a fall from a standing position or less, excluding fractures of the skull, face, fingers, and toes. For morphometric VF assessment, in accordance with standard protocol, a lateral thoraco-lumbar X-ray radiograph was obtained with a tube-to-film distance of 101.6 cm, which included information regarding the positioning of the participants and the radiographic technique used. The radiographs were obtained from the left lateral position, centered at the L1 level. Using Genant’s semi-quantitative method^67^, morphometric vertebral fracture (VF) was diagnosed by three independent radiologists. Disputes regarding the value of a joint were settled by consensus. The VF was determined by evaluating vertebral bodies from the T4 to L4 levels.

### Diagnosis of sarcopenia

The Asian Working Group for Sarcopenia (AWGS) criteria were used to define sarcopenia. The updated 2019 consensus, when compared to the 2014 AWGS criteria, may increase the estimated prevalence of sarcopenia due to an increase in cutoff values for handgrip strength and gait speed. Liu et al.^68^ observed that the significantly associated factors with sarcopenia were not the same when sarcopenia was classified using different criteria. Most factors associated with the 2014 AWGS criteria showed no consistent pattern with the 2019 AWGS, and the validity of the AWGS 2019 consensus requires more research. Since there is no current study on sarcopenia in TDT patients, we decided to investigate its prevalence and associated variables using both the 2014^21^ and 2019^69^ revised AWGS criteria. A DXA whole-body scan was used to determine skeletal muscle mass (Hologic QDR 4500, Inc., Bedford, MA). A skeletal muscle mass index of < 7.0 kg/m^2^ for men and < 5.4 kg/m^2^ for women was used to define muscle mass loss. Handgrip strength was measured using a grip dynamometer (TKK5401®; Takei Scientific Instruments, Tokyo, Japan). The participant was instructed to squeeze the dynamometer with their dominant hand, adducted arm beside the body, and elbow flexed to a 90° angle for 3 s at their maximum force. Handgrip strength was measured twice, with the highest values being recorded. For the 2014 AWGS criteria, low handgrip strength was defined as < 26 kg for men and < 18 kg for women, whereas the 2019 AWGS criteria used < 28 kg for men and < 18 kg for women. Physical performance was determined by the usual 6-m gait speed. Low physical performance was defined as a gait speed cut-off of < 0.8 (the 2014 AWGS criteria) and < 1.0 (the 2019 AWGS criteria) meter per second. Sarcopenia was diagnosed in patients who met the criteria for low muscle mass and either low handgrip strength or low gait speed. Participants with low muscle mass, low handgrip strength, and low gait speed were diagnosed with severe sarcopenia. In this study, overall sarcopenia was defined as the combination of sarcopenia and severe sarcopenia.

### Sample size calculation

Based on a prevalence estimate of the prevalence of osteoporosis in Thai adult with TDT (17.6%)^11^, with a sampling variability of 7%, it was estimated that a sample size of at least 114 was needed for statistical adequacy to estimate the true prevalence of osteoporosis.

### Statistical analyses

Data analysis was performed using SPSS, version 26 for Windows Evaluation Software (SPSS Inc, Chicago, USA). Descriptive statistics were used to categorize and summarize demographic data. Numbers and percentages were used to present gender-specific category data. The Kolmogorov–Smirnov test was used to determine the normality of a continuous data distribution. Data having a normal distribution were presented as means with standard deviations (SD), whereas data with a non-normal distribution were presented as a median with interquartile range (IQR). Comparisons of continuous data between study groups were performed using the unpaired t-test or non-parametric equivalents (Mann–Whitney U test); categorical comparisons were performed using Chi-square or Fisher’s exact test as appropriate. All *P* values were two-sided and considered significant if they were < 0.05.

Logistic regression analysis was performed to calculate the odds ratio (OR) and 95% CI between predictive variables and osteoporosis, sarcopenia, and fragility fracture. The variance inflation factor (VIF) was used to quantify multicollinearity among independent variables. If VIF is > 10, multicollinearity was evaluated. We considered that there was no multicollinearity concern because none of the VIF values exceeded 10. In the multivariable logistic regression (Enter method, SPSS Statistics), all variables with *P* values < 0.20 in the univariate models and known potential confounders were entered. A *P* value < 0.05 was considered statistically significant.

### Supplementary Information

Supplementary Information.

## Data Availability

The datasets generated during and/or analyses during the current study are available from the corresponding author upon reasonable request.
