# Supplementary material for: Association of osteoporosis and sarcopenia with fracture risk in transfusion-dependent thalassemia
Source: Sci Rep. 2023 Sep 29;13:16413. doi: 10.1038/s41598-023-43633-6 (PMC10541420; doi:10.1038/s41598-023-43633-6)
Supplement: Supplementary file 1 — Supplementary Information. [file 41598_2023_43633_MOESM1_ESM.pdf]

Association of Osteoporosis and Sarcopenia with Fracture Risk in Transfusion-Dependent Thalassemia

Suttana Thavonlun[1,2], Natnicha Houngngam[1,2], Kanaungnit Kingpetch[3], Numphung Numkarunarunrote[4], Prangareeya Santisitthanon[1,2], Patinut Buranasupkajorn[1,2], Chatlert Pongchaiyakul[5], Pranee Sutcharitchan[6], Lalita Wattanachanya[1,2*]

[1] Division of Endocrinology and Metabolism, Department of Medicine, Faculty of Medicine, Chulalongkorn University, Bangkok 10330, Thailand

[2] Excellence Center for Diabetes, Hormone, and Metabolism, King Chulalongkorn Memorial Hospital, Bangkok 10330, Thailand

[3] Division of Nuclear Medicine, Department of Radiology, Faculty of Medicine, Chulalongkorn University, Bangkok 10330, Thailand

[4] Division of Diagnostic Radiology, Department of Radiology, Faculty of Medicine, Chulalongkorn University, Bangkok 10330, Thailand

[5] Division of Endocrinology and Metabolism, Department of Medicine, Faculty of Medicine, Khon Kaen University, Khon Kaen 40002, Thailand

[6] Division of Hematology, Department of Medicine, Faculty of Medicine, Chulalongkorn University, Bangkok 10330, Thailand

***Correspondence:**

Associate Professor Lalita Wattanachanya, MD

Division of Endocrinology and Metabolism, Department of Medicine, Faculty of Medicine, Chulalongkorn University, and Excellence Center for Diabetes, Hormone, and Metabolism, King Chulalongkorn Memorial Hospital, Bangkok 10330, Thailand

Tel: + 6622564000, Fax: + 6626525347

Email: lalita_md@yahoo.com

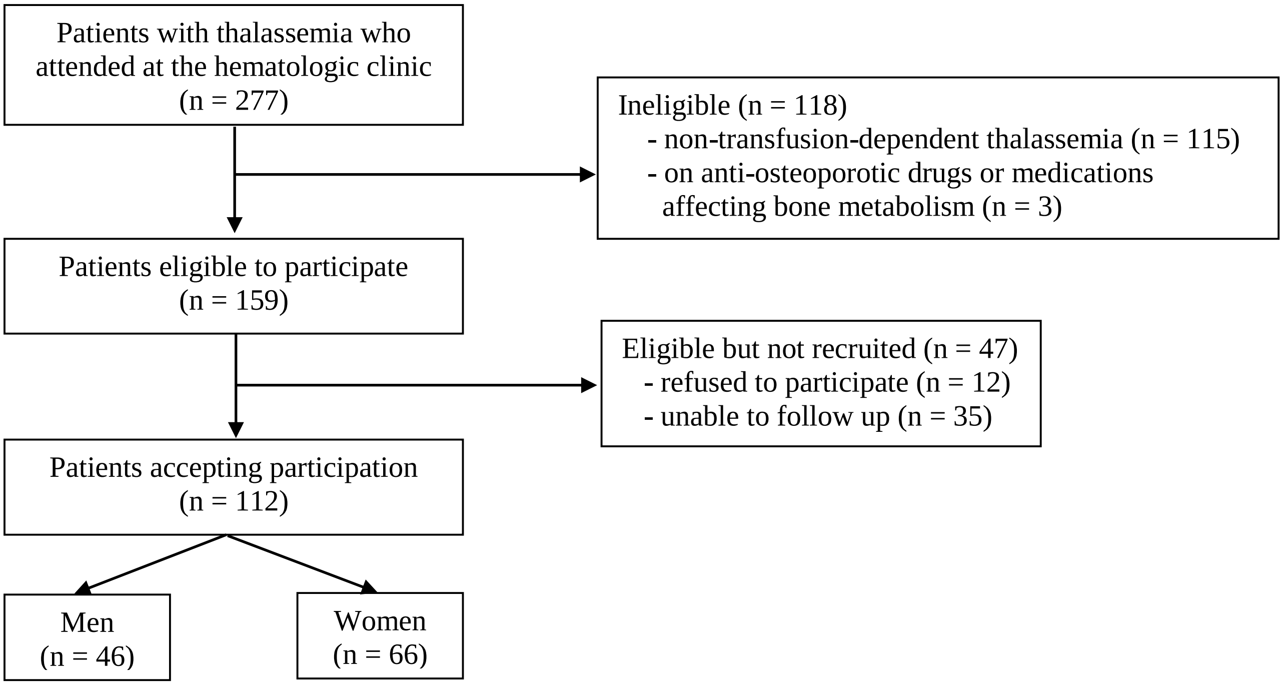

Supplementary figure S1. Study recruitment flowchart. During the study period of August 2020 to November 2020, 277 patients with thalassemia were attended at the hematologic clinic, and 159 patients (57.4%) were eligible to participate in the study. Out of 159 eligible participants, 112 (70.4%) were included in the study. The reason for being eligible but not included was patient refusal to provide consent or unable to follow up.

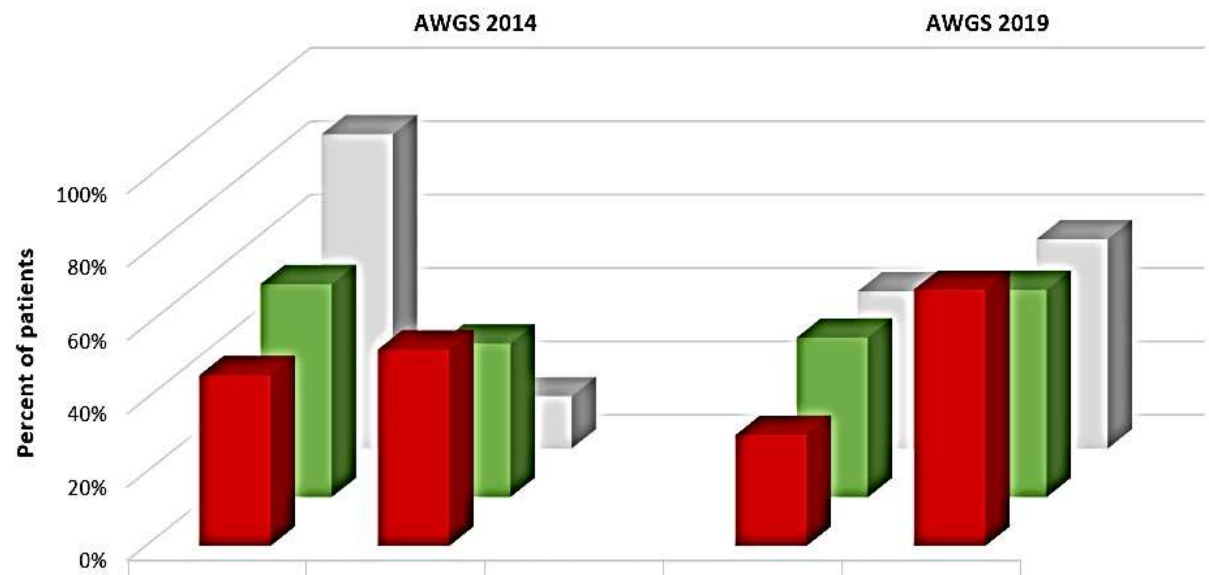

	No sarcopenia (n=62)	Sarcopenia (n=50)		No sarcopenia (n=43)	Sarcopenia (n=69)
Osteoporosis (n=43)	46.5%	53.5%		30.2%	69.8%
Osteopenia (n=62)	58.1%	41.9%		43.5%	56.5%
Normal BMD (n=7)	85.7%	14.3%		42.9%	57.1%

Supplementary figure S2. Prevalence of sarcopenia based on the 2014 and 2019 AWGS criteria and classification of osteoporosis.

Variables	Univariate analysis		Multivariate analysis	
	Odds ratio (95% CI)	*P* value	Odds ratio (95% CI)	*P* value
Age per 10 years	1.81 (1.28, 2.58)	0.001	1.43 (0.82, 2.51)	0.210
Female sex	1.29 (0.59, 2.83)	0.512		
BMI per 5 kg/m^2	0.40 (0.19, 0.83)	0.014	0.29 (0.12, 0.72)	0.007
Hypogonadal stage	4.95 (2.12, 11.59)	<0.001	3.72 (1.09, 12.74)	0.036
History of falls in the past year	11.03 (1.28, 95.09)	0.029	4.48 (0.41, 49.16)	0.219
Current smoking	1.07 (0.172, 6.69)	0.940		
Alcohol drinking > 1 unit/day	2.08 (0.59, 7.27)	0.254		
Splenectomy	1.05 (0.49, 2.28)	0.893		
Iron chelating agents				
- Deferiprone	0.76 (0.35, 1.63)	0.481		
- Deferasirox	1.44 (0.61, 3.39)	0.409		
Pre transfusion Hb, g/dL	1.00 (0.76, 1.33)	0.981		
Ferritin per 1,000 ug/L	1.18 (1.03, 1.36)	0.018	1.09 (0.89, 1.33)	0.384
25 (OH)D, ng/mL	0.99 (0.94, 1.05)	0.801		
Diabetes mellitus	0.31 (0.03, 2.70)	0.286		
CTX, ng/mL	2.63 (0.89, 7.82)	0.081	1.78 (0.14, 23.05)	0.658
P1NP, ng/mL	1.01 (0.99, 1.02)	0.083	1.01 (0.99, 1.02)	0.316
IGF-1, ng/mL	0.98 (0.97, 0.99)	0.004	0.99 (0.98, 1.01)	0.287
Sarcopenia stage (the 2014 AWGS)				
- Sarcopenia	0.98 (0.45, 2.15)	0.960		
- Severe sarcopenia	2.78 (0.91, 8.47)	0.072	1.49 (0.32, 7.02)	0.608

Supplemental table S1. Factors associated with osteoporosis. Logistic regression was used to assess factors associated with osteoporosis. BMI, body mass index; Hb, hemoglobin; 25(OH)D, 25-hydroxyvitamin D; IGF-1, insulin-like growth factor 1; CTX, C-terminal cross-linking telopeptide of type I collagen; P1NP, procollagen type 1 N-terminal propeptide; AWGS, Asian Working Group for Sarcopenia.
